# Complete Genome Sequence of Xanthomonas campestris pv. *campestris* SB80, a Race 4 Strain Isolated from White Head Cabbage in Turkey

**DOI:** 10.1128/mra.00022-22

**Published:** 2022-02-22

**Authors:** Songül Erken Meral, Shaheen Bibi, Carlos Andrés Díaz Rodríguez, Jelena Menković, Adriana J. Bernal, Ralf Koebnik

**Affiliations:** a Department of Plant Health, Black Sea Agricultural Research Institute, Samsun, Turkey; b Department of Plant Pathology and Environmental Microbiology, Pennsylvania State University, University Park, Pennsylvania, USA; c Department of Biological Sciences, Universidad de Los Andes, Bogotá, Colombia; d University of Belgrade, Faculty of Agriculture, Belgrade, Serbia; e Plant Health Institute of Montpellier, University of Montpellier, CIRAD, INRAE, Institut Agro, IRD, Montpellier, France; University of Arizona

## Abstract

Here, we report the complete genome sequence of the race 4 strain Xanthomonas campestris pv. *campestris* SB80, which was isolated from a symptomatic white head cabbage leaf in Samsun Province, Turkey, in 2019. The genome consists of a circular chromosome (5,129,762 bp) with a G+C content of 64.98%, for which 4,159 putative protein-coding genes, 2 rRNA operons, 54 tRNAs, and 86 noncoding RNAs (ncRNAs) were predicted.

## ANNOUNCEMENT

Bacteria of the species Xanthomonas campestris cause black rot, which is the bacterial disease that causes the most devastation to *Brassicaceae* family plants worldwide. The pathovar X. campestris pv. *campestris* has been divided into 11 races based on interactions with a differential set of *Brassica* cultivars, with races 1 and 4 being the most prevalent and destructive (1, 2). Only one draft genome sequence of a race 4 strain, isolated in Chile in 2001, is available at NCBI GenBank (3).

X. campestris pv. *campestris* strain SB80 was isolated from a symptomatic leaf of white head cabbage (Brassica oleracea var. *capitata*) growing in a field in Samsun Province, Turkey, in 2019, as described (4). The race of strain SB80 was determined by evaluating the reactions of various *Brassica* sp. genotypes (compatible, Miracle F1, SxD1, Wirosa F1; incompatible, FBLM2, PIC1, Seven Top Turnip, COB60, Just Right Hybrid Turnip) (1). For DNA isolation, a single colony was grown at 28°C on PSA medium (0.5% peptone, 2% sucrose, 1.5% agar) for 24 h. Bacteria were then resuspended in 10 mM MgCl_2_ and diluted to an optical density at 600 nm of 1.0. Cells from 2 mL of this suspension were harvested by centrifugation, washed once with 10 mM MgCl_2_, and genomic DNA was isolated using the Genomic-tip 100/G protocol (Qiagen, Hilden, Germany) according to the manufacturer’s instructions.

For library construction and sequencing, performed by OhmX.bio (Ghent, Belgium), 1 μg DNA was mechanically fragmented using g-TUBE devices (Covaris, Woburn, MA) at approximately 13 kb. The sequencing library was prepared using the ligation sequencing kit (SQK-LSK110) and the native barcode expansion (PCR-free) pack (EXP-NBD114) based on the manufacturer’s protocol (ONT, Oxford, UK). The samples were sequenced on a GridION R9.4 flow cell for a total of 3 days. Bases were called using MinKNOW v21.10.8. The demultiplexed sequence reads (34,944; *N*_50_, 14,461 bp) were provided by OhmX.bio as FASTQ files.

Adapter sequences were trimmed from the reads using Porechop v0.2.1 (5). The raw reads were checked for quality using NanoFilt (6). The sequences were assembled using Flye v2.9 (7). Default parameters were used for all software unless otherwise specified. Closer inspection revealed issues with homopolymeric nucleotide runs, some of which were manually changed to match the high-quality reference genome sequences for strains ATCC 33913 and 8004 (8, 9). In addition to a large contig of 5.1 Mbp, corresponding to the circular chromosome, the Flye assembly resulted in a second contig of 23 kb, which was almost identical to a region in the large contig and did not encode typical plasmid-associated genes, suggesting an assembly artifact. Notably, this 23-kb region contained a perfect tandem duplication of 1,792 bp in the chromosome but not in the smaller contig. Again, comparison with the two reference genomes prompted us to delete the smaller contig from the assembly and to remove one copy of the duplication in the chromosome.

Assembly and polishing yielded one circular chromosome of 5,129,762 bp with a typical G+C content of 64.98%, corresponding to 136× sequence coverage. The chromosome was annotated using GeneMarkS-2+ (10), as implemented in the NCBI Prokaryotic Genome Annotation Pipeline (http://www.ncbi.nlm.nih.gov/genome/annotation_prok/), which predicted a total of 4,520 genes, including 4,159 coding genes, 215 pseudogenes, 86 noncoding RNAs (ncRNAs), 54 tRNAs, and 2 rRNA operons (5S, 16S, 23S).

This genome sequence for X. campestris from Turkey will facilitate the identification of race-specific factors in X. campestris pv. *campestris* and thus contribute to the development and employment of resistant cabbage cultivars. Interestingly, this strain does not contain an endogenous plasmid, as the other sequenced race 4 strain does. Calculation of genome-wide average nucleotide identities demonstrates that both sequenced race 4 strains belong to two different clades of X. campestris pv. *campestris* (11, 12).

### Data availability.

The genome sequence and raw sequencing reads for strain SB80 were deposited under GenBank accession number CP089952, BioProject accession number PRJNA785926, BioSample accession number SAMN23597367, and SRA accession number SRR17407536.
